# Exploration of the renoprotective effect of Yi-Shen-Hua-Shi granules on db/db mice and the mechanism of podocyte apoptosis based on the GRP78/CHOP signaling pathway

**DOI:** 10.3389/fphar.2025.1586333

**Published:** 2025-09-12

**Authors:** Yanmo Cai, Yunhua Liu, Sitong Wang, Ge Jin, Kaidong Zhou, Xin Zhou, Xinxue Zhang, Min Zhang, Zongjiang Zhao

**Affiliations:** ^1^ International Acupuncture and Moxibustion Innovation Institute, School of Acupuncture-Moxibustion and Tuina, Beijing University of Chinese Medicine, Beijing, China; ^2^ Beijing University of Chinese Medicine, Beijing, China; ^3^ Beijing Chaoyang Hospital, Capital Medical University, Beijing, China

**Keywords:** diabetic kidney disease, endoplasmic reticulum stress, podocyte damage, apoptosis, Yi-Shen-Hua-Shi

## Abstract

**Objective:**

Yi-Shen-Hua-Shi (YSHS) granules are a widely utilized Chinese medicine formula for treating diabetic kidney disease (DKD). Although their effectiveness in treating DKD is established, the precise regulatory mechanism remains unclear. We aimed to explore the potential targets and mechanisms of action of YSHS in delaying DKD progression through network pharmacology and experimental validation.

**Methods:**

Network pharmacology was employed to identify the potential targets and signaling pathways of YSHS in treating DKD, which was hypothesized to be associated with endoplasmic reticulum stress and apoptosis, and these predictions were validated through animal and cellular experiments. Following a 12-week YSHS intervention in db/db mice, assessments were conducted on blood glucose, lipid levels, renal function indices (24-h urinary protein, blood glucose, serum creatinine, blood urea, and urinary albumin-to-creatinine ratio), and kidney pathology. Apoptosis in mouse podocyte clone-5 (MPC-5) cells was assessed using TUNEL labeling. The expression levels of GRP78, PERK, p-PERK, CHOP, Bcl-2, Bax, and Nephrin proteins and mRNAs in mouse kidney tissues and MPC-5 cells were evaluated by immunohistochemistry, Western blotting, and real-time PCR.

**Results:**

YSHS significantly improved the general status of db/db mice, with a significant reduction in body mass, renal function indices, total cholesterol, triglycerides, and low-density lipoprotein. Pathological staining showed reduced renal tissue damage in mice, and electron microscopy revealed reduced pedunculopontine fusion and basement membrane thickening. YSHS decreased GRP78, PERK, p-PERK, CHOP, and Bax proteins and mRNA levels in renal tissues and MPC-5 cells (*p* < 0.05 and *p* < 0.01) while increasing the expression level of Nephrin protein and Bcl-2 mRNA (*p* < 0.05 and *p* < 0.01).

**Conclusion:**

YSHS inhibited podocyte apoptosis, protected the glomerular filtration barrier, attenuated proteinuria, and improved renal function indices by activating the GRP78/CHOP signaling pathway in the kidneys and the *in vitro* cultured podocytes of db/db mice.

## Graphical Abstract

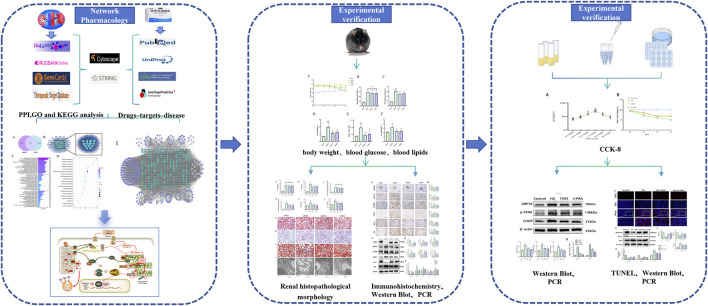

## Highlights

• YSHS granules decrease urinary protein levels, enhance renal function, and slow disease progression in db/db mice.
• YSHS granules can modulate the GRP78/CHOP pathway to attenuate endoplasmic reticulum stress injury in kidney tissue cells of db/db mice.
• Serum containing YSHS reduces apoptosis in podocytes caused by high glucose levels.

## 1 Introduction

Diabetic kidney disease (DKD) is a serious complication of diabetes mellitus, affecting approximately 25%–40% of individuals with diabetes. It is also a leading cause of chronic kidney disease and end-stage renal disease (Alicic et al., 2017; Thomas et al., 2015). DKD progresses slowly, and long-term medication is often associated with drug side effects and resistance (Anders et al., 2018). Therefore, exploring effective methods to prevent and treat DKD is a primary focus of current research. Traditional Chinese medicine (TCM) is distinguished by its complex interactions involving multiple components, targets, and pathways. Some TCM single herbs and compound formulas can effectively regulate blood glucose, improve renal microcirculation, reduce proteinuria, and resist renal fibrosis in patients with DKD. Their mechanisms of action include regulating endoplasmic reticulum stress (ERS), exhibiting anti-inflammatory and antioxidant effects, inhibiting cell death, and ameliorating podocyte damage (Chen et al., 2022; Shen et al., 2024). ERS plays a crucial role in DKD pathogenesis and progression by sustaining cellular homeostasis and adapting to internal environmental changes. Excessive ERS exacerbates oxidative stress, inflammation, apoptosis, and pyroptosis, thereby aggravating kidney injury (Jung and Yoo, 2022). The endoplasmic reticulum manages adaptive and apoptotic ERS via the molecular chaperone BiP/Grp78 and the three unfolded protein response (UPR) pathways when stimulated by stress-inducing factors (Fan et al., 2017; Hetz and Papa, 2018). GRP78 and CHOP are recognized markers of ERS and UPR activation. ERS is closely associated with three transmembrane proteins. Under stress, the protein kinase R-like endoplasmic reticulum kinase (PERK) is hyperactivated and undergoes autophosphorylation. Phosphorylated PERK (p-PERK) further upregulates the expression of CHOP, the C/EBP homolog, which is also known as GADD153. CHOP is a transcription factor that represses the expression of Bcl-2, which encodes an antiapoptotic gene, thereby activating the apoptotic program (Oakes and Papa, 2015).

Yi-Shen-Hua-Shi (YSHS) granules, approved by China’s State Food and Drug Administration, are used to treat kidney diseases such as DKD, chronic glomerulonephritis, and immunoglobulin A nephropathy. Originating from the TCM formula “Sheng Yang Yi Wei Tang” by master Li Dongyuan, YSHS comprises 16 herbs (Table 1). Wang Dongyuan showed that YSHS can improve the lipid metabolism in patients with DKD, reduce renal damage, and lower urinary protein levels (Wang and Liu, 2018). Additionally, YSHS can regulate intestinal flora dysbiosis and reduce proteinuria in patients with CKD (Dong et al., 2024). YSHS has been shown to lower serum or blood urea nitrogen (BUN) and serum creatinine (SCr) levels, decrease urinary 24-h PRO and UAER, alleviate oxidative stress, and improve renal function in patients with DKD (Li et al., 2020). Our previous studies revealed that YSHS reduces the activation of monocyte chemoattractant protein-1 (MCP-1)/C-C chemokine receptor type 2 (CCR2) through the p38 mitogen-activated protein kinase (MAPK) signaling pathway, attenuates the tubular damage caused by high glucose, and protects tubular epithelial cells (Zhou et al., 2025). It has been shown that p38 MAPK phosphorylates and activates the stress-induced transcription factor C/EBP homology protein (CHOP), and it also affects its target gene GRP78 (Krupkova et al., 2018; Wang and Ron, 1996).

**TABLE 1:** YSHS herbal ingredients list.

CHN Pinyin name	Latin name (scientific name)	English name (Pharmacopeia of China 2020)
Renshen	*Panax ginseng* C. A. Mey	Ginseng Radix Et Rhizoma
Huang Qi	*Astragalus mongholicus* Bunge	Astragali Radix
Baizhu	*Atractylodes macrocephala* Koidz	Atractylodis Rhizoma
Fuling	Poria Cocos (Schw.) Wolf	Poria
Zexie	Alisma plantago-aquatica Linn	Alismatis Rhizoma
Banxia	*Pinellia ternata* (Thunb.) Makino	Pinelliae Rhizoma Praeparatum Cum Alumine
Qiang Huo	*Hansenia weberbaueriana* (Fedde ex H.Wolff) Pimenov & Kljuykov	Notopterygii Rhizoma et Radix
Duhuo	*Angelica biserrata* (R.H. Shan and C.Q. Yuan) C.Q. Yuan and R.H. Shan	Angelicae Pubescentis Radix
Fangfeng	*Saposhnikovia divaricata* (Turcz.) Schischk	Saposhnikoviae Radix
Chaihu	Bupleurum chinense DC.	Bupleuri Radix
Huanglian	*Coptis chinensis* Franch.	Coptidis Rhizoma
Baishao	*Paeonia lactiflora* Pall.	Paeoniae Radix Alba
Chenpi	*Citrus reticulata* Blanco.	Citri Reticulatae Pericarpium
Gancao	Glycyrrhiza uralensis Fisch. ex DC.	Glycyrrhizae Radix et Rhizoma
Dazao	*Ziziphus jujuba* Mill.	Jujubae Fructus
Shengjiang	*Zingiber officinale* Rosc.	Zingiberis Rhizoma Recens

In this study, we used network pharmacology to investigate YSHS’s active ingredients, potential targets, and pathways for DKD treatment. However, the specific mechanisms through which YSHS exerts its effects on DKD remain unclear. To address this, we further investigated the involvement of YSHS in regulating the ERS GRP78/CHOP signaling pathway, using both *in vivo* animal models and *in vitro* cellular experiments. This approach sought to clarify the mechanism by which YSHS prevents and treats DKD.

## 2 Materials and methods

### 2.1 Network pharmacology analysis

According to the reported literature, a total of 105 chemical components were detected in YSHS using high-performance liquid chromatography coupled with quadrupole time-of-flight mass spectrometry (Agilent 1290 Infinity II LC/6545 Q-TOF, Agilent Technologies, United States) (Chan et al., 2021). To identify the therapeutic targets of YSHS for DKD treatment, we utilized the TCM Systems Pharmacology (TCMSP) Database (https://www.tcmsp-e.com/load_intro.php?id=43) to analyze the chemical components of the herbal medicines in the YSHS formula. The screening criteria included an oral bioavailability of ≥30% and drug-likeness of ≥0.18. We gathered and structured all the chemical constituents of YSHS according to these parameters. The target names obtained from the screening were validated using the UniProt database (https://www.uniprot.org/) to identify potential targets of the active ingredients of YSHS, and we compiled a target information dataset by searching five databases—Online Mendelian Inheritance in Man (https://www.omim.org/), GeneCards (https://www.genecards.org/), DrugBank (https://go.drugbank.com/), Therapeutic Target Database (https://db.idrblab.net/ttd/), and DisGeNET (https://disgenet.com/)—using the following keywords: “diabetic nephropathy,” “diabetic kidney disease,” and “diabetic nephrosis.” This search yielded a list of genes associated with DKD, which were selected as the candidate targets. Genes from the five databases were integrated, and the target gene dataset for DKD was obtained after removing duplicates. The Venn online tool (https://www.bioinformatics.com.cn/) was used to identify overlapping targets between DKD-related genes and YSHS active ingredient targets. The intersecting gene dataset from the Venn diagrams identified the potential target genes of YSHS for DKD treatment.

The STRING database (https://string-db.org) was utilized to screen for “Homo sapiens” with a minimum interaction confidence score of 0.400. The interaction score was defined as a confidence score of 0.400 or higher, and the potential targets of YSHS for DKD treatment were analyzed using protein–protein interaction (PPI) and visualized using Cytoscape (version 3.10.1, Cytoscape Consortium).

### 2.2 Functional enrichment analysis

Metascape database (https://metascape.org/gp/index.html) was used to perform Gene Ontology (GO) functional analysis and Kyoto Encyclopedia of Genes and Genomes (KEGG) pathway enrichment of the potential targets. The targets were analyzed using Metascape with personalized settings, specifying “sapiens” as the organism, a significance threshold of *p* < 0.01, a minimum count of three, and an enrichment factor greater than 1.5. GO functional analysis was performed, covering bovine process, cellular component, and molecular function, along with KEGG pathway enrichment. The results were visualized using bar charts and bubble plots.

### 2.3 Molecular docking

The 2D structures of the key components of YSHS were downloaded from the PubChem database (https://www.pubchem.ncbi.nlm.nih.gov/) in the Protein Data Bank (PDB) format and optimized using Chem3D (version 14.0.0.17, PerkinElmer Informatics, Inc.). In the PDB, the crystal structures of the target proteins containing the corresponding ligands and resolved by X-ray diffraction were screened according to the principle of optimal resolution, and the key target proteins were selected. The water molecules and the original small molecule ligands in the structure were removed, and hydrogen atoms were added using AutoDock Tools (ADT) (version 1.5.7, Molecular Graphics Laboratory (MGL), The Scripps Research Institute). We set up the rotatable bonds of the compounds and determined the docking box size based on the receptor protein to ensure full coverage of its three-dimensional space. Virtual docking of the YSHS active ingredient and target proteins was performed using ADT. The conformation of the compound corresponding to the best binding free energy for each protein was selected, and finally, the three-dimensional model was drawn using PyMOL (version 3.1.3, Schrödinger, LLC).

### 2.4 Drug preparation

YSHS granules (Guangzhou Kangchen Pharmaceutical, GuoYaoZhunZi-Z: 20090250, Lot: 20210802, Guangzhou, China) and dapagliflozin (10 mg; NH3205, Cambridge, United Kingdom) were purchased from AstraZeneca.

### 2.5 Animal grouping and drug administration

Thirty 7-week-old male db/db mice and ten db/m mice, all of which were of a specific pathogen-free grade, were purchased from Changzhou Cavins Laboratory Animal Co. Ltd., license no.: SCXK(SU)2021-0013. The animals were housed in a room at the Beijing University of Traditional Chinese Medicine (BUCM) under controlled conditions: temperature of 24 °C–26 °C, 50%–60% relative humidity, and with unrestricted access to food and water. The experimental animals were handled in strict compliance with the requirements of the Experimental Animal Ethics Committee of BUCM (ethics no.: the Beijing University of Traditional Chinese Medicine; 2022082403-3181). Following a 5-week acclimatization and feeding period, fasting blood glucose was assessed from samples taken at the tail tip of the mice, and 24-h urine was collected to measure urinary albumin/creatinine (UACR) levels. The enrollment criteria included fasting blood glucose levels of at least 11.1 mmol/L and UACR of at least 3 mg/mmol. The db/m mice served as normal controls, representing a healthy, nondiabetic baseline group. The db/db mice, used to model diabetes, were divided into three groups: the untreated model group, the dapagliflozin group, and the YSHS group. Each group contained 10 mice, and stratified randomization was based on body mass to ensure balanced group distribution. Dapagliflozin was administered by gavage at a dose of 1.6 mg/kg. The dose of YSHS for the mice was converted from the human clinical dose using the body surface area formula and combined with the results of the pretest, and 4.7 g/kg was selected as the optimal experimental dose (Supplementary material 1). Mice in both the normal and model groups were gavaged with equal volumes of deionized water once daily for 12 weeks.

### 2.6 General condition and the urine and blood biochemistry of mice

The general condition of mice in each group, such as the mental state, hair, water intake, food intake, and activity, was observed, and the body mass of the mice was recorded every 2 weeks. Before and after 12 weeks of drug administration, the mice in each group were fasted, their tail tips were pricked with disposable blood collection needles, and tail-tip blood glucose was detected with a glucometer. Urine was collected before and after 12 weeks of drug administration using a mouse metabolic cage, and the total 24-h urine volume was recorded. The 24-h urine protein content of the mice was calculated using the BCA Protein Assay Kit (Beyotime, Shanghai, China, P0012) after extracting the collected urine protein using a liquid protein extraction kit (LABLEAD, Beijing, China, B5000). After 12 weeks of drug administration, the mice were anaesthetized by injecting 0.1% sodium pentobarbital solution (50 mg/kg). Blood was collected by removing the eyeballs, and the samples were allowed to stand for 2 h. After centrifugation, renal function indices, including SCr, blood urea (UREA), triglyceride (TG), low-density lipoprotein cholesterol (LDL), and total cholesterol (TC), were measured using an automatic biochemistry analyzer (Myriad, Shenzhen, China, BS-420).

### 2.7 Renal histopathological analysis

Kidney tissues from the mice in each group were fixed in 10% formalin, routinely dehydrated, paraffin-embedded, sectioned (4 μm), and stained with hematoxylin and eosin (HE; Solarbio, Beijing, China, G1080), periodic acid–Schiff stain (PAS; Regenerative Biology, Zhejiang, China, 0331A22), and Mallory stain (Solarbio, G1355). After sealing the sections, the relevant tissues were observed under a light microscope (Nikon, Tokyo, Japan, Ti2-ER01A) for structural and pathological changes.

### 2.8 Transmission electron microscopy

Renal tissue samples (1 mm^3^) from each group were fixed in 2.5% glutaraldehyde osmium acid and prepared for electron microscope samples. The samples were sectioned using an ultrathin microscope (Leica, Wetzlar, Germany, UC7), and a transmission electron microscope (TEM, Hitachi, Osaka, Japan, H-7650) was used to observe the ultrastructure of various groups of renal tissues.

### 2.9 Immunohistochemical observation of mouse kidney tissue protein expression

Paraffin sections from each group were routinely deparaffinized and rehydrated in water. Sections were rinsed in phosphate-buffered saline (PBS) containing 0.01% Triton and incubated at 37 °C for 10 min. The samples were rinsed in PBS containing H_2_O_2_-CH_3_OH and incubated at 22 °C ± 1 °C for 10 min. Following PBS washing, tissue sections were subjected to antigen retrieval by incubation in citrate buffer (pH 6.0) with microwave irradiation at 98 °C for 15 min, followed by gradual cooling to room temperature (22 °C ± 1 °C). Sections were rinsed in PBS, blocked with 10% goat serum (Solarbio, SL038) at 37 °C for 2 h, and then incubated overnight at 4 °C with the following titrated primary antibodies: GRP78 (1:75, Rabbit Polyclonal, GTX113340, Gene Tex, United States), phospho-PERK (1:200, GTX00673, Gene Tex, United States), CHOP (1:200, Mouse Monoclonal, Thermo Fisher Scientific, MA1-250, United States), Nephrin (1:400, Rabbit Monoclonal, ab216341, Abcam, United Kingdom), Bax (1:65, Rabbit Polyclonal, GTX109683, Gene Tex, United States), and Bcl-2 (1:65, Rabbit Polyclonal, GTX100064, Gene Tex, United States). The sections were then rinsed with PBS. The corresponding species-specific secondary antibodies, goat anti-rabbit IgG H&L (HRP) (1:1,000, ab6721, Abcam, United Kingdom) or goat anti-mouse IgG H&L (HRP) (1:1,000, ab6789, Abcam, United Kingdom), were incubated at 37 °C for 30 min. The sections were rinsed again with PBS, followed by the application of DAB for color development. Hematoxylin was used for re-staining, followed by rinsing with running water to return the blue hue. Finally, the sections were sealed with neutral gum after undergoing gradient alcohol dehydration. Photographs were taken under a light microscope, and brownish-yellow particles were regarded as positive expression. ImageJ 1.53 (NIH, United States) was used to find the statistics of the area that was positively expressed.

### 2.10 Western blot detection of mouse kidney tissue protein expression

Mouse kidney tissues stored at −80 °C were thawed, and approximately 100 mg of tissue was cut and minced. The tissues were washed with saline before being homogenized in radioimmunoprecipitation assay (RIPA) protein lysate (LABLEAD, Beijing, China, R1091) containing protease inhibitor (LABLEAD, C0101) and phosphatase inhibitor (LABLEAD, C0104) to extract proteins. After homogenization, the sample underwent lysis on ice for 30 min, followed by centrifugation at 12,000 rpm for 15 min. The upper protein clear liquid was collected and determined using the BCA Kit, and protein denaturation was performed by adding 5× sampling buffer (Accellerase, Beijing, China, 037A16995) in a metal bath for 10 min. A 10% SDS-PAGE (sodium dodecyl sulfate–polyacrylamide gel electrophoresis) was prepared, with a protein upload volume of 30 μg. After electrophoresis (100 V) and electrotransfer (100 V), a 5% skimmed milk powder solution was applied for blocking at 22 °C ± 1 °C for 2 h. Antibodies GRP78 (1:2,000), phospho-PERK (1:1,000), PERK (1:1,000), CHOP (1:1,000), Nephrin (1:5,000), Bax (1:2,000), Bcl-2 (1:1,000), and β-actin (1:2000) were incubated overnight at 4 °C. Following Tris-buffered saline with Tween (TBST) rinsing, species-specific horseradish peroxidase (HRP)-conjugated secondary antibodies (1:10,000) were added and incubated at 22 °C ± 1 °C for 2 h. The blots were rinsed again with TBST and visualized using an electrogenerated chemiluminescence (ECL) solution (Beyotime, P0018S), and then they were exposed on the ChemiDoc MP Imaging System (Bio-Rad, United States, 17001402). ImageJ was used to analyze the target bands.

### 2.11 Real-time polymerase chain reaction (PCR) detection of messenger ribonucleic acid (mRNA) expression in mouse kidney tissue

Mouse kidney tissue (100 mg) was weighed and sheared on ice. One milliliter of precooled TRIzol lysate (LABLEAD, Beijing, China, R1000) was added to mouse kidney tissue mRNA from each group, and the RNA concentration and purity were assessed using NanoDrop Lite Plus (Thermo Fisher Scientific, Waltham, MA, United States). Reverse transcription followed instructions on the kit, and RNA expression levels were quantified via real-time fluorescence PCR using the Eastep® RT Master Mix Kit (Promega, Shanghai, China, LS 2062) with the following conditions: 95 °C for 30 s, followed by 40 cycles, 95 °C for 15 s, and 60 °C for 50 s. Primers used for RT-PCR were purchased from Sangon Biotech (Shanghai, China) (Table 2). The relative expression of the genomes was determined using the 2^−ΔΔCT^ method.

**TABLE 2:** Primer sequence.

Gene name	Primer sequence (5’ - 3′)	bp
GRP78	F:AAATGACAAAACCGCCTGACACCTG	331
	R:GGGCCTCCACTTCCATAGAGTTTGCT	
CHOP	F:CGCTCTCCAGATTCCAGTCA	141
	R:GTTCTCCTGCTCCTTCTCCTT	
Nephrin	F:CCCAACACTGGAAGAGGTGT	200
	R:CTGGTCGTAGATTCCCCTTG	
Bcl-2	F:CTGTGGATGACTGAGTACC	127
	R:GAGACAGCCAGGAGAAAT	
Bax	F:ATCCAAGACCAGGGTGGCT	198
	R:CCTTCCCCCATTCATCCCAG	
β-Actin	F:TCCTGTGGCATCCACGAAACT	315
	R:GAAGCATTTGCGGTGGACGAT	

### 2.12 Preparation of the drug-containing serum

Six male SPF-grade SD rats aged 6–8 weeks and weighing approximately 200 g ± 20 g were purchased from Spearfish (Beijing) Biotechnology Co., Ltd.: SCXK (Beijing) 2019-0010. The rats were divided into a blank group and a serum-containing group. The serum-containing group received YSHS via gastric gavage at a dose of 3.5 g/kg, which corresponds to seven times the clinical dose for humans (Nair et al., 2018). In the blank group, water was administered to the rats by gavage at an identical dose. After 1 week of continuous administration, the drug-containing serums were inactivated, filtered, and stored at −80 °C.

### 2.13 Cell culture and subleased drug delivery

The mouse podocyte clone-5 (MPC-5) cells, with conditional immortality, were provided by BeNa Culture Collection (Beijing, China, BNCC342021). The conditionally immortalized MPC-5 mouse podocyte cell line (originally established by Dr. Peter Mundel) was used as an *in vitro* model of podocyte injury. MPC-5 cells were resuscitated and cultured in RPMI1640 medium (Servicebio, G4530) supplemented with 10% fetal bovine serum (FBS; Servicebio, G8003) containing 100 ug/l penicillin and streptomycin. The cells were maintained at 33 °C in a 5% CO_2_ incubator. The cell growth status was observed, and then the cells were transferred to interferon (IFN)-γ-free medium and cultured at 37 °C in a 5% CO_2_ incubator for 10–15 days. Maturely differentiated MPC-5 cells were then inoculated into petri dishes for culture. Cells at approximately 50% confluence were synchronized in the G_0_ phase by incubating them in serum-free medium for 24 h. Cells were then divided into four groups: 1) normal (5% normal group rat serum +3% FBS + normal sugar medium [5.5 mmol/L]+ mannitol [24.5 mmol/L]), 2) high glucose (5% normal group rat serum +3% FBS + high glucose medium [30 mmol/L]), 3) YSHS (5% YSHS group rats with serum +3% FBS + high glucose medium [30 mmol/L]), and 4) 4-phenylbutyric acid (4-PBA; Sigma-Aldrich, St. Louis, MO, United States, SML0309) (5% normal group rat serum +3% FBS +2.5 mmol/L 4-PBA for 1 h before continuing with the high glucose medium) (Cao et al., 2016).

### 2.14 Cell activity assay

Mature differentiated MPC-5 cells were seeded in 96-well plates (5,000 cells per well). Once cell growth reached approximately 50%, the cells were incubated for 12, 24, 36, and 48 h, followed by a 2-h incubation with the Cell Counting Kit (CCK)-8 solution (Absin, Shanghai, China, 20230805) per well in an incubator. Cell viability was estimated by measuring the absorbance (450 nm) with a Spectra Max i3x Multi-Mode Microplate Reader (i3x-1000, Molecular Devices). The optimal survival rate was chosen for further experiments.

### 2.15 TUNEL staining

MPC-5 cells were cultured in 24-well plates and fixed with 10% formaldehyde for 15 min following 48-h treatment with high glucose and drug-containing serum. Subsequently, 100 μL of TUNEL mix (TdT + FITC-labeled dUTP) was applied as per the manufacturer’s instructions and incubated for 60 min at 37 °C in a dark environment. The plates were then sealed with an anti-fluorescence quencher and imaged using fluorescence microscopy (Nikon, Tokyo, Japan, Ti2-EFLA).

### 2.16 Cell protein and mRNA extraction

After 48 h of cell culture, the lysate was added, and the cells in the culture dish were scraped and collected. Proteins and mRNA were extracted for Western blot and PCR, respectively, using the previously outlined experimental procedures.

### 2.17 Statistical analysis

Statistical analyses were performed using IBM SPSS Statistics software (version 26.0; IBM Corporation, Armonk, NY, United States). One-way analysis of variance (ANOVA) was used for comparisons, followed by the least significant difference (LSD) test for pairwise comparisons. When ANOVA assumptions were not met, Dunnett’s T3 method was used. A significance level of α = 0.05 was used, where *p* < 0.05 denotes statistically significant differences. The statistical results were graphically displayed using GraphPad Prism 10.0.

## 3 Results

### 3.1 Network pharmacology analysis of YSHS in DKD treatment

**FIGURE 1:**
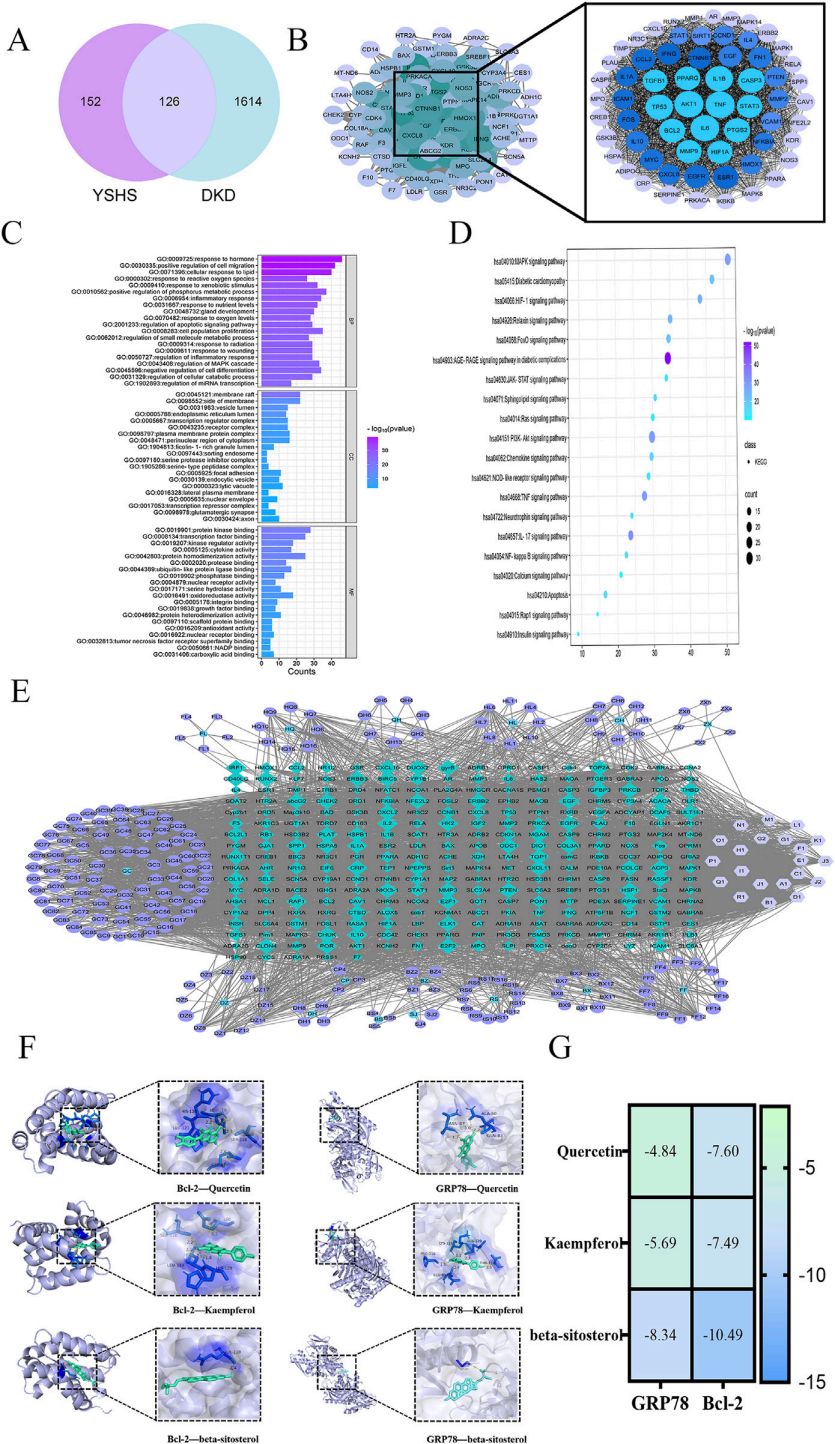
Network pharmacology analysis of YSHS for DKD. **(A)** Drug target and disease target intersection Venn diagram analysis of YSHS and DKD. **(B)** Network of protein interactions of key targets. **(C)** GO enrichment bar graph of key target genes. **(D)** KEGG pathway enrichment bubble diagram of key target genes. **(E)** Active ingredient and disease target of YSHS against DKD. **(F)** Molecular docking visualization. **(G)** Molecular docking binding energy heatmap.

After eliminating duplicates through database and literature review, we identified 205 active ingredients of YSHS and 278 targets from the TCMSP and UniProt databases (Table 3). The 1,740 disease targets were selected for intersection and mapped with the 278 target genes corresponding to the active ingredients to obtain 126 core targets (Figure 1A). A PPI network comprising the 126 key targets was constructed. After excluding discrete protein targets, the network consisted of 126 nodes and 3,067 edges, representing the interconnections among the proteins. Using the CytoHubba tool within the Cytoscape plug-in, the topological properties of the PPI network were analyzed to determine and rank the node gene degrees. The top 10 genes identified were as follows: AKT1, TNF, IL-6, IL-1B, TP53, Caspase-3, PTGS2, BCL2, EGFR, and PPARG (Figure 1B). The potential targets of YSHS for DKD treatment were analyzed using Metascape software. GO enrichment analysis results indicated involvement in hormonal and inflammatory responses, apoptotic signaling regulation, and microRNA (miRNA) transcriptional regulation. The molecular functions included binding to protein kinases, transcription factors, nuclear receptors, and phosphatases. The cellular components included membrane rafts, endoplasmic reticulum, endosomal lumen, plasma membrane microvesicles, and endocytosis vesicles (Figure 1C). The KEGG-enriched signaling pathways included the AGE-RAGE, PI3K-Akt, MAPK, HIF-1, FoxO, and NF-kappa B pathways. The YSHS-active ingredient-target-of-action-DKD graph was plotted, featuring 498 nodes and 4,590 edges. The main active ingredients were β-sitosterol, quercetin, kaempferol, stigmasterol, and iso-europregnanol (Figure 1D). YSHS targets multiple signaling pathways in DKD treatment, with endoplasmic reticulum-related targets such as GRP78 and Bcl-2 potentially playing crucial roles (Figure 1E). The molecular docking results showed that quercetin, kaempferol, and β-sitosterol had good binding sites with the ligand proteins. Through van der Waals force and hydrogen bonding interactions with the ligand proteins, it was found that β-sitosterol had the lowest binding energies to the ligand proteins GRP78 and Bcl-2, which were −8.34 kcal/mol and −10.49 kcal/mol, respectively. Quercetin showed a weaker binding to GRP78 (−4.84 kcal/mol) but a stronger binding to Bcl-2 of −7.60 kcal/mol. The binding energy of kaempferol to Bcl-2 was −7.49 kcal/mol, which was similar to that of quercetin (Figures 1F, G).

**TABLE 3:** Sequence of active ingredient primers for pharmacological screening of the YSHS–DKD network.

Node	MOL ID	Molecule name	Node	MOL ID	Molecule name
A1	MOL000359	Sitosterol	DZ18	MOL000096	(−)-Catechin
C1	MOL000358	β-Sitosterol	DH1	MOL004780	Angelicone
D1	MOL000422	Kaempferol	G1	MOL001941	Ammidin
BS4	MOL001919	(3S,5R,8R,9R,10S,14S)-3,17-Dihydroxy-4,4,8,10,14-pentamethyl-2,3,5,6,7,9-hexahydro-1H-cyclopenta [a]phenanthrene-15,16-dione	DH3	MOL004777	Angelol D
BS5	MOL001924	Paeoniflorin	G2	MOL001942	Isoimperatorin
F1	MOL000211	Mairin	DH6	MOL004778	[(1R,2R)-2,3-Dihydroxy-1-(7-methoxy-2-oxochromen-6-yl)-3-methylbutyl] (Z)-2-methylbut-2-enoate
L1	MOL000492	(+)-Catechin	R1	MOL004792	Nodakenin
BS8	MOL001918	Paeoniflorgenone	DH8	MOL003608	O-Acetylcolumbianetin
BZ2	MOL000022	14-Acetyl-12-senecioyl-2E,8Z,10E-atractylentriol	FF1	MOL000011	(2R,3R)-3-(4-Hydroxy-3-methoxy-phenyl)-5-methoxy-2-methylol-2,3-dihydropyrano[5,6-h][1,4]benzodioxin-9-one
I1	MOL000033	(3S,8S,9S,10R,13R,14S,17R)-10,13-Dimethyl-17-[(2R,5S)-5-propan-2-yloctan-2-yl]-2,3,4,7,8,9,11,12,14.15,16,17-dodecahydro-1H-cyclopenta [a]phenanthren-3-ol	FF2	MOL011730	11-Hydroxy-sec-o-beta-d-glucosylhamaudol_qt
BZ1	MOL000049	3β-Acetoxy-atractylone	FF3	MOL011732	Anomalin
BZ4	MOL000072	8β-Ethoxy atractylenolide Ⅲ	FF4	MOL011737	Divaricatacid
BX1	MOL001755	24-Ethylcholest-4-en-3-one	FF5	MOL011740	Divaricatol
BX2	MOL002670	Cavidine	FF7	MOL011747	Ledebouriellol
BX3	MOL002714	Baicalein	FF8	MOL011749	Phelloptorin
P1	MOL002776	Baicalin	FF9	MOL011753	5-O-Methylvisamminol
E1	MOL000449	Stigmasterol	O1	MOL002644	Phellopterin
BX7	MOL005030	Gondoic acid	FF12	MOL000173	Wogonin
BX8	MOL000519	Coniferin	FF14	MOL001494	Mandenol
BX9	MOL006936	10,13-Eicosadienoic	FF16	MOL003588	Prangenidin
BX10	MOL006957	(3S,6S)-3-(Benzyl)-6-(4-hydroxybenzyl)piperazine-2,5-quinone	FF17	MOL007514	Methyl icosa-11,14-dienoate
BX11	MOL003578	Cycloartenol	FF18	MOL013077	Decursin
BX12	MOL006967	β-D-Ribofuranoside, xanthine-9	FL1	MOL000273	(2R)-2-[(3S,5R,10S,13R,14R,16R,17R)-3,16-Dihydroxy-4,4,10,13,14-pentamethyl-2,3,5,6,12.15,16,17-octahydro-1H-cyclopenta [a]phenanthren-17-yl]-6-methylhept-5-enoic acid
CH1	MOL001645	Linoleyl acetate	FL2	MOL000275	Trametenolic acid
H1	MOL000354	Isorhamnetin	FL3	MOL000279	Cerevisterol
CH6	MOL004598	3.5,6,7-Tetramethoxy-2-(3,4,5-trimethoxyphenyl)chromone	FL4	MOL000282	Ergosta-7,22E-dien-3beta-ol
CH7	MOL004609	Areapillin	FL5	MOL000283	Ergosterol peroxide
CH8	MOL013187	Cubebin	K1	MOL000296	Hederagenin
CH9	MOL004624	Longikaurin A	GC1	MOL001484	Inermine
CH10	MOL004653	(+)-Anomalin	GC2	MOL001792	DFV
CH11	MOL004718	α-Spinasterol	GC3	MOL002311	Glycyrol
CH12	MOL000490	Petunidin	J1	MOL000239	Jaranol
B1	MOL000098	Quercetin	GC5	MOL002565	Medicarpin
CP2	MOL005828	Nobiletin	GC9	MOL003656	Lupiwighteone
CP3	MOL005815	Citromitin	GC10	MOL003896	7-Methoxy-2-methyl isoflavone
CP4	MOL005100	5,7-Dihydroxy-2-(3-hydroxy-4-methoxyphenyl)chroman-4-one	J2	MOL000392	Formononetin
Q1	MOL004328	Naringenin	J3	MOL000417	Calycosin
DZ1	MOL012921	Stepharine	GC15	MOL004805	(2S)-2-[4-Hydroxy-3-(3-methylbut-2-enyl)phenyl]-8,8-dimethyl-2,3-dihydropyrano [2,3-f]chromen-4-one
DZ2	MOL012946	Zizyphus saponin I_qt	GC16	MOL004806	Euchrenone
DZ3	MOL012976	Coumestrol	GC17	MOL004808	Glyasperin B
DZ4	MOL012981	Daechuine S7	GC18	MOL004810	Glyasperin F
DZ5	MOL012986	Jujubasaponin V_qt	GC19	MOL004811	Glyasperin C
DZ6	MOL012992	Mauritine D	GC20	MOL004814	Isotrifoliol
N1	MOL001454	Berberine	GC21	MOL004815	(E)-1-(2,4-Dihydroxyphenyl)-3-(2,2-dimethylchromen-6-yl)prop-2-en-1-one
DZ8	MOL001522	(S)-Coclaurine	GC22	MOL004820	Kanzonols W
DZ12	MOL004350	Ruvoside_qt	GC23	MOL004824	(2S)-6-(2,4-Dihydroxyphenyl)-2-(2-hydroxypropan-2-yl)-4-methoxy-2,3-dihydrofuro [3,2-g]chromen-7-one
DZ14	MOL000627	Stepholidine	GC24	MOL004827	Semilicoisoflavone B
DZ15	MOL007213	Nuciferin	GC25	MOL004828	Glepidotin A
M1	MOL000787	Fumarine	GC26	MOL004829	Glepidotin B
DZ17	MOL002773	β-Carotene	GC27	MOL004833	Phaseolinisoflavan
GC28	MOL004835	Glypallichalcone	GC78	MOL005003	Licoagrocarpin
GC29	MOL004838	8-(6-Hydroxy-2-benzofuranyl)-2,2-dimethyl-5-chromenol	GC79	MOL005007	Glyasperin M
GC30	MOL004841	Licochalcone B	GC80	MOL005008	Glycyrrhiza flavonol A
GC31	MOL004848	Licochalcone G	GC81	MOL005012	Licoagroisoflavone
GC32	MOL004849	3-(2,4-Dihydroxyphenyl)-8-(1,1-dimethylprop-2-enyl)-7-hydroxy-5-methoxy-coumarin	GC82	MOL005016	Odoratin
GC33	MOL004855	Licoricone	GC83	MOL005017	Phaseol
GC34	MOL004856	Gancaonin A	GC84	MOL005018	Xambioona
GC35	MOL004857	Gancaonin B	GC85	MOL005020	Dehydroglyasperin C
GC36	MOL004863	3-(3,4-Dihydroxyphenyl)-5,7-dihydroxy-8-(3-methylbut-2-enyl)chromone	HL1	MOL002668	Worenine
GC37	MOL004864	5,7-Dihydroxy-3-(4-methoxyphenyl)-8-(3-methylbut-2-enyl)chromone	HL2	MOL001458	Coptisine
GC38	MOL004866	2-(3,4-Dihydroxyphenyl)-5,7-dihydroxy-6-(3-methylbut-2-enyl)chromone	HL4	MOL002904	Berlambine
GC39	MOL004879	Glycyrin	HL6	MOL002897	Epiberberine
GC40	MOL004882	Licocoumarone	HL7	MOL002907	Corchoroside A_qt
GC41	MOL004883	Licoisoflavone	HL8	MOL002903	(R)-Canadine
GC42	MOL004884	Licoisoflavone B	HL10	MOL002894	Berberrubine
GC43	MOL004885	Licoisoflavanone	HL11	MOL000785	Palmatine
GC44	MOL004891	Shinpterocarpin	HQ6	MOL000371	3,9-Di-O-methylnissolin
GC45	MOL004898	(E)-3-[3,4-Dihydroxy-5-(3-methylbut-2-enyl)phenyl]-1-(2,4-dihydroxyphenyl)prop-2-en-1-one	HQ7	MOL000378	7-O-Methylisomucronulatol
GC46	MOL004903	Liquiritin	HQ8	MOL000379	9,10-Dimethoxypterocarpan-3-O-β-D-glucoside
GC47	MOL004904	Licopyranocoumarin	HQ9	MOL000380	(6aR,11aR)-9,10-Dimethoxy-6a,11a-dihydro-6H-benzofurano [3,2-c]chromen-3-ol
GC48	MOL004907	Glyzaglabrin	HQ10	MOL000387	Bifendate
GC49	MOL004908	Glabridin	HQ14	MOL000433	FA
GC50	MOL004910	Glabranin	HQ15	MOL000439	Isomucronulatol-7.2′-di-O-glucosiole
GC51	MOL004911	Glabrene	HQ16	MOL000442	1,7-Dihydroxy-3,9-dimethoxy pterocarpene
GC52	MOL004912	Glabrone	QH2	MOL011963	8-Geranoxy-5-methoxypsoralen
GC53	MOL004914	1,3-Dihydroxy-8,9-dimethoxy-6-benzofurano [3,2-c]chromenone	QH3	MOL011969	Demethylfuropinnarin
GC54	MOL004915	Eurycarpin A	QH4	MOL011971	Diversoside_qt
GC55	MOL004924	(−)-Medicocarpin	QH5	MOL011975	Notoptol
GC56	MOL004935	Sigmoidin-B	QH6	MOL001951	Bergaptin
GC57	MOL004941	(2R)-7-Hydroxy-2-(4-hydroxyphenyl)chroman-4-one	QH7	MOL001956	Cnidilin
GC58	MOL004945	(2S)-7-Hydroxy-2-(4-hydroxyphenyl)-8-(3-methylbut-2-enyl)chroman-4-one	QH13	MOL002881	Diosmetin
GC59	MOL004948	Isoglycyrol	RS5	MOL002879	Diop
GC60	MOL004949	Isolicoflavonol	RS6	MOL003648	Inermin
GC61	MOL004957	HMO	RS7	MOL005308	Aposiopolamine
GC62	MOL004959	1-Methoxyphaseollidin	RS8	MOL005317	Deoxyharringtonine
GC63	MOL004961	Quercetin der	RS9	MOL005318	Dianthramine
GC64	MOL004966	3′-Hydroxy-4′-O-methylglabridin	RS10	MOL005320	Arachidonate
GC65	MOL000497	Licochalcone a	RS11	MOL005321	Frutinone A
GC66	MOL004974	3′-Methoxyglabridin	RS12	MOL005344	Ginsenoside rh2
GC67	MOL004978	2-[(3R)-8,8-Dimethyl-3,4-dihydro-2H-pyrano [6,5-f]chromen-3-yl]-5-methoxyphenol	RS13	MOL005348	Ginsenoside-Rh4_qt
GC68	MOL004980	Inflacoumarin A	RS14	MOL005356	Girinimbin
GC69	MOL004985	Icos-5-enoic acid	RS15	MOL005376	Panaxadiol
GC70	MOL004988	Kanzonol F	RS16	MOL005384	Suchilactone
GC71	MOL004990	7.2′,4′-Trihydroxy-5-methoxy-3-arylcoumarin	RS17	MOL005399	Alexandrin_qt
GC72	MOL004991	7-Acetoxy-2-methylisoflavone	SJ2	MOL006129	6-Methylgingediacetate2
GC73	MOL004993	8-Prenylated eriodictyol	SJ4	MOL001771	Poriferast-5-en-3beta-ol
GC74	MOL004996	Gadelaidic acid	ZX2	MOL000831	Alisol B monoacetate
GC75	MOL000500	Vestitol	ZX3	MOL000849	16β-Methoxyalisol B monoacetate
GC76	MOL005000	Gancaonin G	ZX4	MOL000853	Alisol B
GC77	MOL005001	Gancaonin H	ZX5	MOL000856	Alisol C monoacetate
ZX7	MOL000862	[(1S,3R)-1-[(2R)-3,3-Dimethyloxiran-2-yl]-3-[(5R,8S,9S,10S,11S,14R)-11-hydroxy-4,4,8,10,14-pentamethyl-3-oxo-1,2,5,6,7,9,11,12,15,16-decahydrocyclopenta [a]phenanthren-17-yl]butyl] acetate	ZX6	MOL002464	1-Monolinolein

### 3.2 General pharmacodynamic effects of YSHS in db/db mice

**FIGURE 2:**
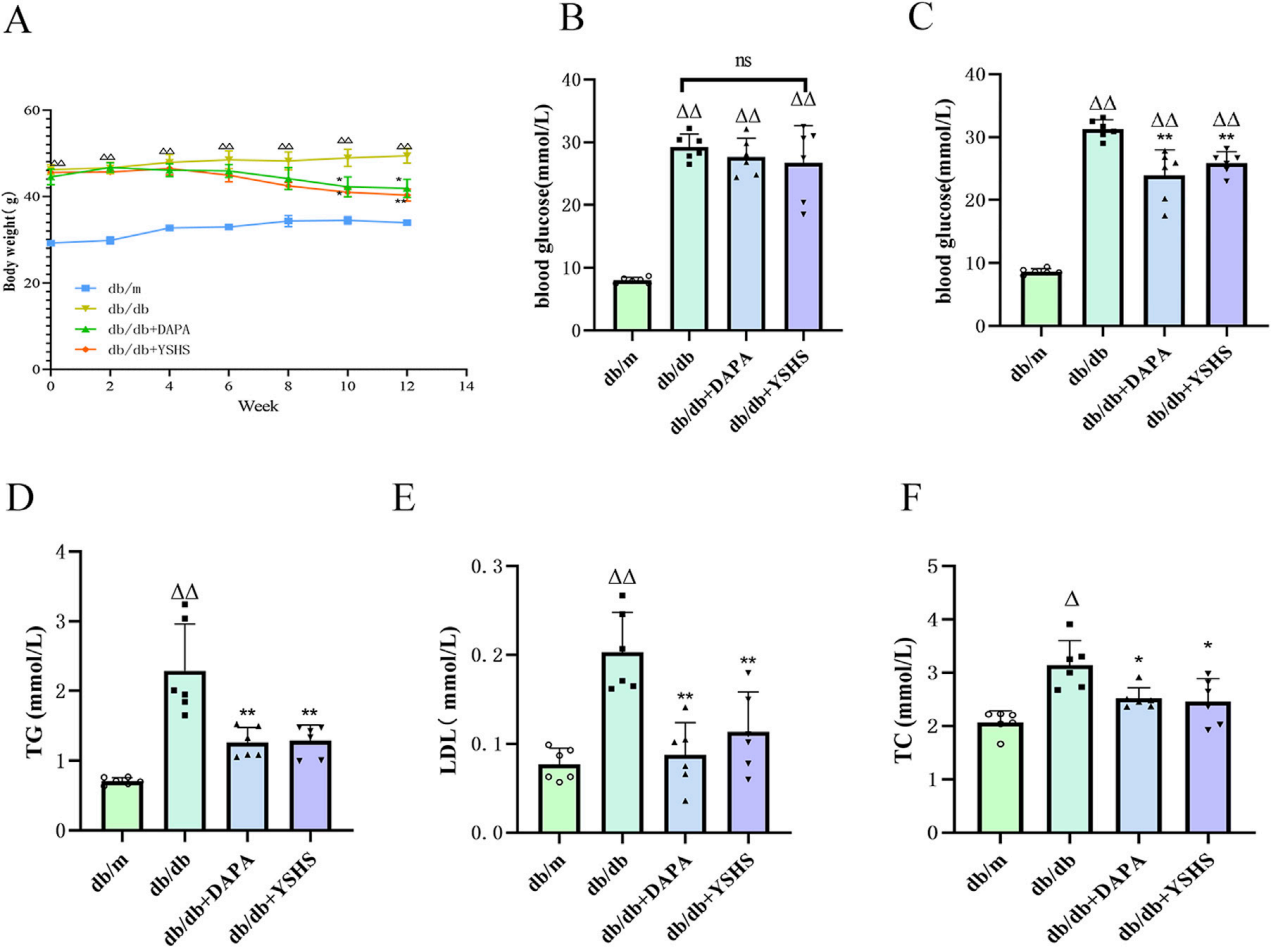
YSHS improves DKD-related indices in mice. **(A)** Body weight of mice. **(B, C)** Blood glucose before and after dosing. **(D, E, F)** Improvement of blood lipids in mice after drug administration. All data were expressed as means ± SEM (N = 6). Statistical analysis was performed using one-way ANOVA followed by Dunnett’s *post hoc* test for multiple comparisons. Compared to the normal group, ^△^ *p* < 0.05 and ^△△^ *p* < 0.01; compared to the model group, ^*^ *p* < 0.05 and ^**^ *p* < 0.01. ns: no statistical difference.

The db/db mice appeared depressed, with significantly reduced activity. Their fur lost its luster and appeared dull, while fat accumulated in the groin and axilla. Moreover, the levels of feeding, drinking, and urination were notably elevated compared with those in the normal group. Post-administration, the overall condition of the mice improved in both the dapagliflozin and YSHS groups. After 12 weeks of intervention, body weight, blood glucose, LDL, TC, and total cholesterol levels were significantly lower in the model group of mice in the YSHS group and the dapagliflozin group than in the model group (p < 0.05 and p < 0.01, respectively), suggesting that YSHS was effective in decreasing the blood glucose and lipids in DKD mice (Figure 2).

### 3.3 YSHS attenuates histopathological damage and protects renal function in db/db mice

**FIGURE 3:**
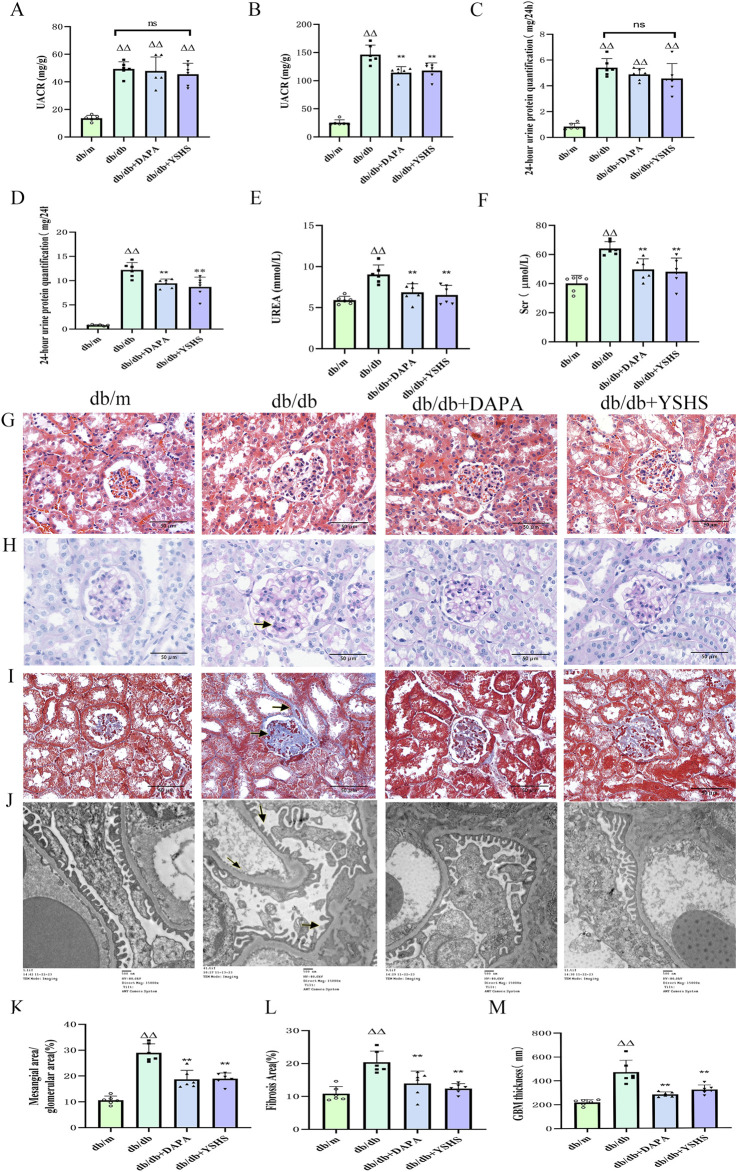
YSHS improves renal function and histopathologic morphology in mice. **(A, B)** UACR before and after drug administration. **(C–F)** Reduction of 24-h urinary protein, SCr, and UREA after drug administration. **(G)** Images of HE staining of the kidney tissues in the different groups (scale bar, 50 μm). **(H)** Images of PAS staining of the kidney tissues in the different groups (scale bar, 50 μm). **(I)** Images of Mallory staining of the kidney tissues in the different groups (scale bar, 50 μm). **(J)** TEM analysis of kidney tissues in the different groups of mice (scale bar, 00 nm). **(K)** Mesangial area/glomerular area. **(L)** Relative fibrotic area (%) based on Mallory staining. **(M)** GBM thickness. All data were expressed as means ± SEM (N = 6). Statistical analysis was performed using one-way ANOVA followed by Dunnett’s *post hoc* test for multiple comparisons. Compared to the normal group, ^△^ *p* < 0.05 and ^△△^ *p* < 0.01; compared to the model group, ^*^ *p* < 0.05 and ^**^ *p* < 0.01. ns: no statistical difference.

The model group showed significantly elevated levels of UACR, 24-h urinary protein, SCr, and UREA compared to the control group (*p* < 0.01). The YSHS and dapagliflozin groups showed significantly lower indicators than the model group (*p* < 0.01), suggesting a beneficial regulatory effect of YSHS on renal function (Figures 3A–F).

Light microscopy of HE-stained sections showed normal glomeruli, tubules, and interstitial structures in the control group mice. The model group showed vacuolated tubular epithelial cells and moderate glomerular mesangial cell proliferation in the renal tissues. In contrast, the YSHS and dapagliflozin groups exhibited fewer vacuolated tubular cells and decreased mesangial cell proliferation (Figure 3G). The results of PAS staining showed no extracellular matrix accumulation in the glomeruli of normal mice under a light microscope. In contrast, extracellular matrix accumulation was observed in the glomeruli of mice in the model group. The renal tissue widening in mice was notably less in the YSHS and dapagliflozin groups (Figures 3H, K). Mallory staining revealed that the model group mice exhibited significant collagen fiber proliferation in the glomerular mesangial area and tubular tubulointerstitial area. Treatment with YSHS and dapagliflozin markedly reduced collagen fiber hyperplasia and renal fibrosis (Figures 3I, L).

TEM analysis of mouse glomerular peduncle ultrastructure showed normal basement membrane and peduncle protrusions in the control group. In contrast, the model group demonstrated severe fusion of glomerular peduncle protrusions, segmental thickening of the basement membrane, and obvious pathological alterations. However, after the administration of dapagliflozin and YSHS, the thickness of the basement membrane of the peduncle cells was decreased, and the degree of fusion of the peduncle protrusions was reduced (Figures 3J, M).

### 3.4 YSHS inhibits ERS in the kidney of db/db mice

**FIGURE 4:**
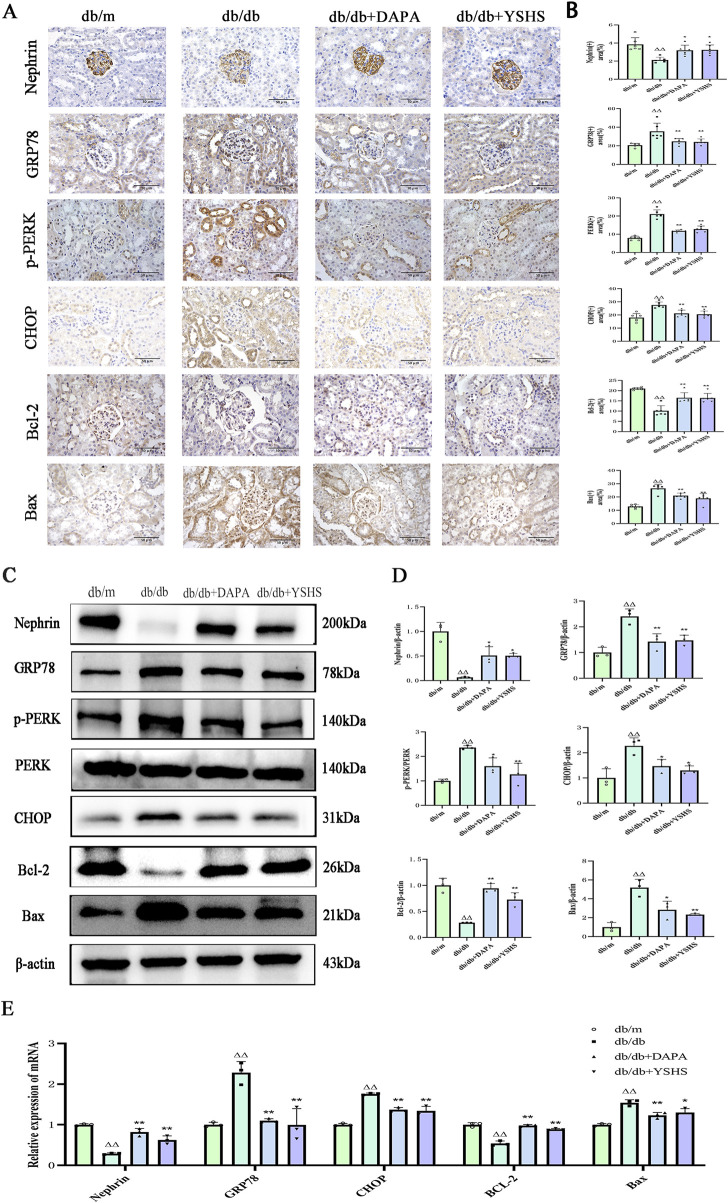
Effect of YSHS on renal ERS levels in DKD mice. Immunohistochemistry of mouse kidney GRP78, CHOP, **(A)** Nephrin, Bcl-2, and Bax (×400, bar = 50 μm). **(B)** Statistical histogram of mouse kidney immunohistochemistry. **(C)** Western blot experiment of YSHS modulation of mouse kidney GRP78, p-PERK, CHOP, Nephrin, Bcl-2, and Bax. **(D)** Statistical graph of mouse kidney Western blot experiment. **(E)** Effect of YSHY on mRNA of GRP78, CHOP, Nephrin, Bcl-2, and Bax in mouse kidney. All data were expressed as means ± SEM (N = 3). Statistical analysis was performed using one-way ANOVA followed by Dunnett’s *post hoc* test for multiple comparisons. Compared to the normal group, ^△^ *p* < 0.05 and ^△△^ *p* < 0.01; compared to the model group, ^*^ *p* < 0.05 and ^**^ *p* < 0.01. ns: no statistical difference.

The results of our immunohistochemistry (Figures 4A, B) and Western blot experiments (Figures 4C, D) showed that the expression of Nephrin and Bcl-2 in the kidneys of mice in the db/db model group was significantly reduced (*p* < 0.01), whereas the intervention of dapagliflozin and YSHS significantly increased their expression levels (*p* < 0.01 and *p* < 0.05). In addition, the expression levels of GRP78, PERK, CHOP, and Bax were significantly elevated in the renal tissues of db/db model mice (*p* < 0.01), whereas both dapagliflozin and YSHS reduced the expression of these markers (*p* < 0.01 and *p* < 0.05).

Real-time PCR analysis showed that the expression levels GRP78, CHOP, and Bax mRNA were significantly upregulated in the kidneys of mice in the model group (*p* < 0.01). YSHS and dapagliflozin significantly reduced their expression (*p* < 0.01 and *p* < 0.05). The real-time PCR results for Nephrin and Bcl-2 mRNA aligned with the immunohistochemistry and Western blot findings, showing statistical significance with a *p*-value of <0.05 (Figure 4E).

### 3.5 YSHS improves the viability of high glucose-injured MPC-5 cells

**FIGURE 5:**
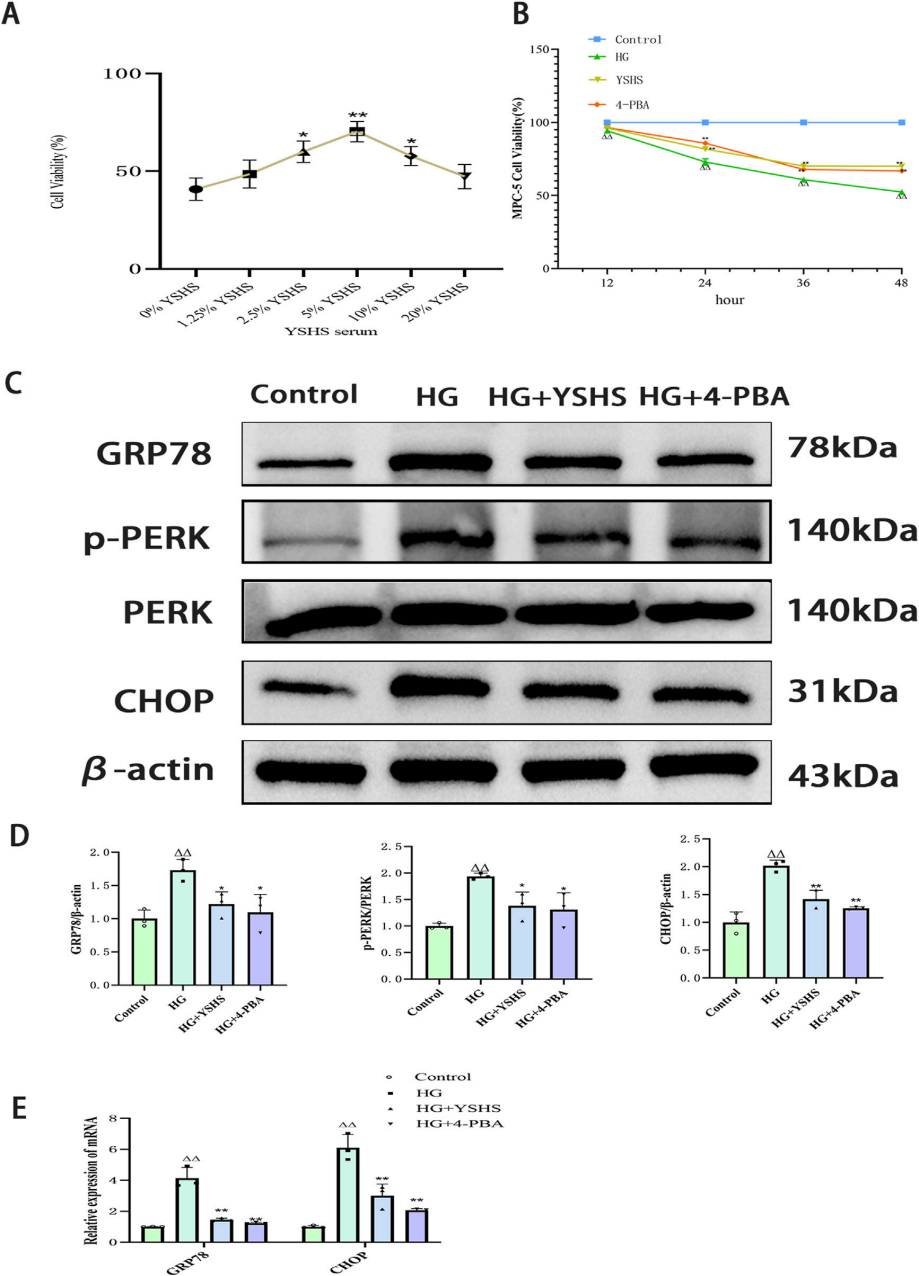
Effect of YSHS on ERS levels in high glucose-induced MPC-5 podocytes. **(A)** Effect of different YSHS on the activity of MPC-5 cells. **(B)** Effect of YSHS on endoplasmic stress network of MPC-5 induced by high glucose. Detection of MPC-5 cell viability at different time periods. **(C)** Western blot experiments of GRP78, p-PERK, and CHOP of MPC-5. **(D)** Statistical graph of Western blot experiments of MPC-5. **(E)** Effect of YSHS on mRNA of GRP78 and CHOP of MPC-5. All data were expressed as the means ± SEM (N = 3). Statistical analysis was performed using one-way ANOVA followed by Dunnett’s *post hoc* test for multiple comparisons. Compared to the normal group, ^△^ *p* < 0.05 and ^△△^ *p* < 0.01; compared to the model group, ^*^ *p* < 0.05 and ^**^ *p* < 0.01. ns: no statistical difference.

CCK8 experiments demonstrated a significant reduction in mouse podocyte viability after 12 h of high glucose stimulation, whereas MPC-5 cell viability significantly decreased after 24, 36, and 48 h of continuous high glucose exposure (*p* < 0.01). YSHS-containing serum and 4-PBA improved MPC-5 cell viability after 24 h and significantly increased viability compared with the high-sugar group (*p* < 0.01). Therefore, in the subsequent experiments, 48 h of high glucose stimulation was chosen as the time for high glucose-induced podocyte injury (Figures 5A, B).

### 3.6 YSHS inhibits high glucose-induced ERS in MPC-5 cells

Western blot showed that the expression levels of GRP78, p-PERK, and CHOP were significantly elevated in podocytes after high glucose intervention (*p* < 0.01 and *p* < 0.05). The expression levels of GRP78, p-PERK, and CHOP were significantly reduced in the podocytes of both the 4-PBA and YSHS groups compared with the model group (*p* < 0.01 and *p* < 0.05) (Figures 5C, D).

Real-time PCR analysis revealed that high glucose significantly increased GRP78 and CHOP expression in MPC-5 cells (*p* < 0.01). Additionally, both GRP78 and CHOP mRNA expression were downregulated in MPC-5 cells treated with YSHS and 4-PBA (*p* < 0.05) (Figure 5E).

### 3.7 YSHS attenuates high glucose-induced apoptosis in MPC-5 cells

**FIGURE 6:**
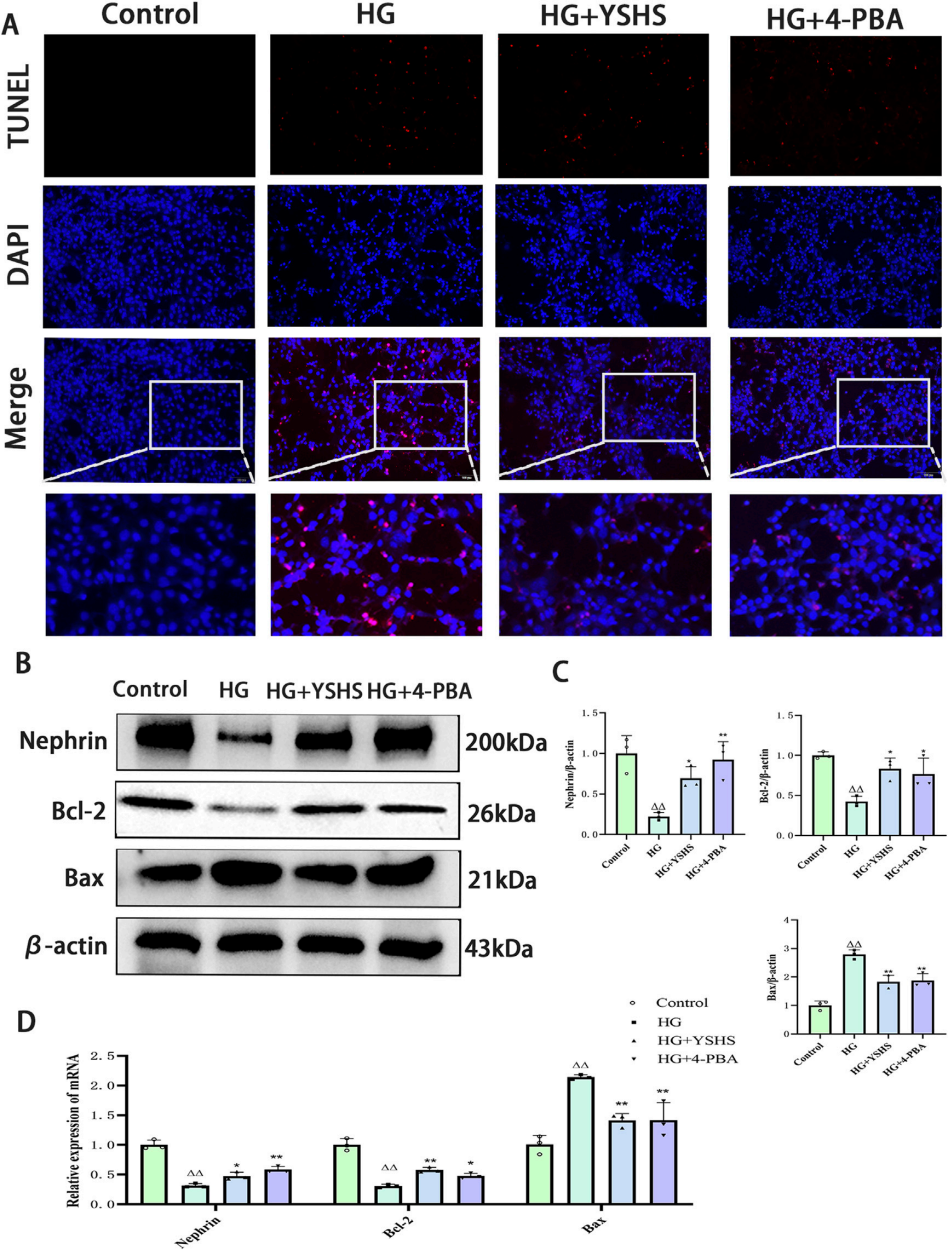
Effect of YSHS on high glucose-induced apoptosis in MPC-5 podocytes. **(A)** Image of TUNEL staining results. **(B)** Western blot experiments of Nephrin, Bcl-2, and Bax of MPC-5. **(C)** Statistical graph of Western blot experiments of MPC-5. **(D)** Effect of YSHS on mRNA of Nephrin, Bcl-2, and Bax of MPC-5. All data were expressed as means ± SEM (N = 3). Statistical analysis was performed using one-way ANOVA followed by Dunnett’s *post hoc* test for multiple comparisons. Compared to the normal group, ^△^ *p* < 0.05 and ^△△^ *p* < 0.01; compared to the model group, ^*^ *p* < 0.05 and ^**^ *p* < 0.01. ns: no statistical difference.

TUNEL staining revealed a significant increase in apoptotic cells in the MPC-5 cell group exposed to high glucose. In contrast, high glucose-induced apoptosis in MPC-5 cells was reduced under YSHS or 4-PBA intervention (Figure 6A).

Western blot analysis indicated that high glucose notably increased Bax expression while decreasing Bcl-2 and Nephrin levels in MPC-5 cells (*p* < 0.01). YSHS and 4-PBA modulated Bax, Bcl-2, and Nephrin expression in MPC-5 cells, showing decreased Bax and increased Bcl-2 and Nephrin levels compared with the high-glucose group (*p* < 0.01 and *p* < 0.05) (Figures 6B, C).

Real-time PCR analysis indicated that high-glucose conditions significantly increased Bax mRNA expression while decreasing Bcl-2 and Nephrin mRNA levels in MPC-5 cells (*p* < 0.01). Bax mRNA expression was decreased, whereas the expression levels of Bcl-2 and Nephrin mRNA were increased in MPC-5 cells of the YSHS and 4-PBA groups compared with the high-glucose group (*p* < 0.01 and *p* < 0.05) (Figure 6D).

## 4 Discussion

DKD initially presents as microproteinuria, progresses to overt proteinuria and worsening renal impairment, and, in the late stage, leads to severe renal failure and irreversible renal damage. YSHS is an effective TCM for the treatment of DKD. Clinical studies have shown that YSHS granules can suppress SCr, BUN, and urinary protein levels in patients; improve renal injury; and reduce clinical symptoms such as edema and fatigue (Ai et al., 2022; Tan et al., 2022; Xu et al., 2024; Zhao et al., 2019). Although some studies have shown that YSHS attenuates renal inflammation and fibrosis through transforming growth factor beta (TGF-β), bone morphogenetic protein 2 (BMP2)/Smad, and nuclear factor (NF)-κB signaling pathways, the complex multi-targeted molecular mechanisms require further elucidation.

To elucidate the mechanism of action of YSHS, we employed a network pharmacology system to analyze and predict its potential key targets of action for the treatment of DKD. Protein interaction network analysis identified AKT1, TGF-β1, TNF, Caspase-3, BCL2, and GRP78 as the core action targets of YSHS. Meanwhile, KEGG and GO enrichment analyses indicated that the mechanism of action involves the regulation of apoptosis, inflammatory response, MAPK signaling pathway, and ERS. Another of our previous studies had shown that YSHS reduces MCP-1/CCR2 activation in mouse kidney through the p38 MAPK signaling pathway (Zhou et al., 2025). However, p38 MAPK phosphorylates and activates the stress-induced transcription factor CHOP while affecting its target gene GRP78. Therefore, we hypothesized that YSHS prevents and treats diabetic nephropathy primarily by modulating the GRP78/CHOP signaling pathway associated with ERS and ameliorating apoptosis.

Studies have confirmed that ERS is one of the main pathogenic mechanisms of DKD (Yang et al., 2023). Chronic hyperglycemia and proteinuria can trigger the production of large amounts of UPR by renal tissue cells, leading to the development of ERS (Zhang et al., 2023).Glucose-regulated protein 78 (GRP78), which is associated with the ERS, is located in the endoplasmic reticulum membrane of all eukaryotes; it facilitates protein folding and assembly, manages protein mass, and regulates ERS signaling. The accumulation of unfolded proteins causes GRP78 to dissociate from the transmembrane sensor protein kinases PERK, ATF6, and IRE1, thereby activating them and initiating the degradation of endoplasmic reticulum-associated proteins (Ibrahim et al., 2019; Ni et al., 2011). The dissociation of PERK from GRP78 phosphorylates eIF2α, which further activates ATF4 and UPR target genes such as CHOP and GADD34. CHOP, a proapoptotic transcription factor downstream of PERK, regulates genes encoding proteins linked to proliferation, differentiation, and energy metabolism. According to clinical trials, patients with DKD exhibit increased serum levels of GRP78 and CHOP (Ma et al., 2021). Interestingly, the results of our *in vitro* experiments revealed that YSHS downregulated GRP78, p-PERK, and CHOP in renal tissues of DKD, thereby reducing ERS and improving renal function.

In addition, CHOP plays a central role in orchestrating cellular responses to stress and regulates numerous target genes associated with multiple rescue pathways that enhance cell survival (Li et al., 2014). Low levels of ERS activate pro-survival signaling, whereas severe and persistent ERS activates GRP78/CHOP apoptotic signaling (Oyadomari and Mori, 2004; Ron and Habener, 1992). CHOP overexpression transcriptionally represses antiapoptotic gene Bcl-2, thereby promoting apoptosis (Iurlaro and Muñoz-Pinedo, 2016; Zong et al., 2003). One study further confirmed that GRP78 was upregulated and Bcl-2 was downregulated in renal tissues of DKD mice (Zhang et al., 2022). Interestingly, our study also found that YSHS downregulated Bcl-2, upregulated Bax, and attenuated apoptosis in renal tissues, which may be related to the regulation of the upstream gene CHOP by YSHS to improve ERS.

In addition, in animal experiments, we noted that YSHS significantly attenuated podocyte peduncle fusion and podocyte loss in db/db mice and significantly upregulated the expression of Nephrin in the podocytes of renal tissues, which is in line with the findings of Zeng et al. (2016). Podocytes are essential cells that preserve the structure and function of the glomerular filtration barrier. The development of microalbuminuria in patients with DKD is associated with podocyte loss, which is one of the earliest features of DKD to predict its progression (Meyer et al., 1999; Wolf et al., 2005). One study further demonstrated that podocytes in patients with DKD were subjected to high glucose-induced conditions, which allowed morphological and functional alterations, including hypertrophy, loss of pedicle protrusions, apoptosis, and autophagy, implying podocyte injury (Maezawa et al., 2015). Nephrin is a crucial structural protein in the podocytes slit diaphragm, and it is essential for maintaining the podocytes’ structure and the glomerular filtration barrier’s physiological function (Allison, 2021).

To further validate the *in vivo* findings of YSHS, we used MPC-5 mouse foot cells for *in vitro* experiments. Under high glucose-induced conditions, mouse foot cells undergo ERS, leading to upregulation of GRP78 and CHOP, which ultimately triggers apoptosis (Cao et al., 2014). Regarding apoptosis-related protein Bcl-2 as a potential target gene, it has not been reported that YSHS can achieve the mechanism of action of kidney protection by regulating ERS-induced apoptosis. In our experiments, we screened the appropriate concentration of YSHS by using a high glucose-induced ERS injury model of podocytes, and we observed that YSHS could effectively improve the survival rate of podocytes at 48 h by cell activity assay and TUNEL assay. In molecular biology experiments, we used 4-PBA, a potent inhibitor of ERS, as a control; 4-PBA is a molecular chaperone that promotes protein folding and mitigates ERS (Cao et al., 2016), and in high glucose environments, 4-PBA protects the kidney by inhibiting ERS (Upagupta et al., 2017; Li et al., 2023; Carlisle et al., 2014). In our experiments, we also observed that the effect of YSHS was comparable to that of 4-PBA, which downregulated CHOP expression and improved high glucose-induced ERS of podocytes, thereby upregulating the antiapoptotic protein Bcl-2 and downregulating Bax expression, which in turn reduced podocyte apoptosis.

In addition, on further investigating the key active substances of YSHS, we found that quercetin, kaempferol, and β-sitosterol might be the main active components of YSHS in the drug component–target network analysis of network pharmacology. These compounds possess broad-spectrum anti-inflammatory and antioxidant properties. Current evidence suggests that quercetin exerts nephroprotective effects through multiple mechanisms: it increases renal Bcl-2 levels, inhibits CHOP mRNA expression, and attenuates ERS and apoptosis through sirtuin 1 (SIRT1)-dependent deacetylation, thus effectively attenuating cadmium chloride-induced kidney injury in rats. In addition, quercetin has good therapeutic potential for glucosamine-induced apoptosis and inflammation by inhibiting ERS (Alshammari et al., 2021; Cai et al., 2017). Similarly, kaempferol has been shown to protect against liver failure in murine models by modulating the ERS-Grp78-CHOP signaling pathway, thereby inhibiting hepatocyte apoptosis (Wang et al., 2019). β-Sitosterol appears to prevent nonalcoholic steatohepatitis by simultaneously reducing oxidative stress, ERS, and inflammatory responses (Abo-Zaid et al., 2023). In addition, we further verified our conjecture by molecular docking, in which β-sitosterol showed the strongest binding affinity to two key targets, GRP78 and Bcl-2, and quercetin and kaempferol also showed good binding potential to Bcl-2. In summary, these findings strongly suggest that quercetin, kaempferol, and β-sitosterol represent the core pharmacologically active components of YSHS for the prevention and treatment of DKD. However, this is only a preliminary network virtual validation, and future surface plasmon resonance (SPR), microscale thermophoresis (MST), and other experiments are needed to verify the binding activities of these active small molecules with the target proteins.

Although this study demonstrates YSHS’s renoprotective effects through GRP78/CHOP pathway modulation in male db/db mice, several limitations must be acknowledged. The mechanistic actions of YSHS’s active components (quercetin, kaempferol, and β-sitosterol) require deeper investigation, particularly regarding their direct targeting of the ER stress pathways and the causal relationship between ER stress suppression and apoptosis inhibition. Our exclusive focus on the GRP78/CHOP axis leaves other critical ERS branches (ATF6 and XBP1) unexplored, whereas the male-only design—although controlling for hormonal variability—precludes the assessment of sex-specific effects, which is a significant consideration given estrogen’s known regulation of both ER stress and lipid metabolism. Future studies should employ genetic manipulations (e.g., GRP78/CHOP knockdown) and include both sexes to fully elucidate YSHS’s therapeutic mechanisms and translational potential.

## 5 Conclusion

**FIGURE 7:**
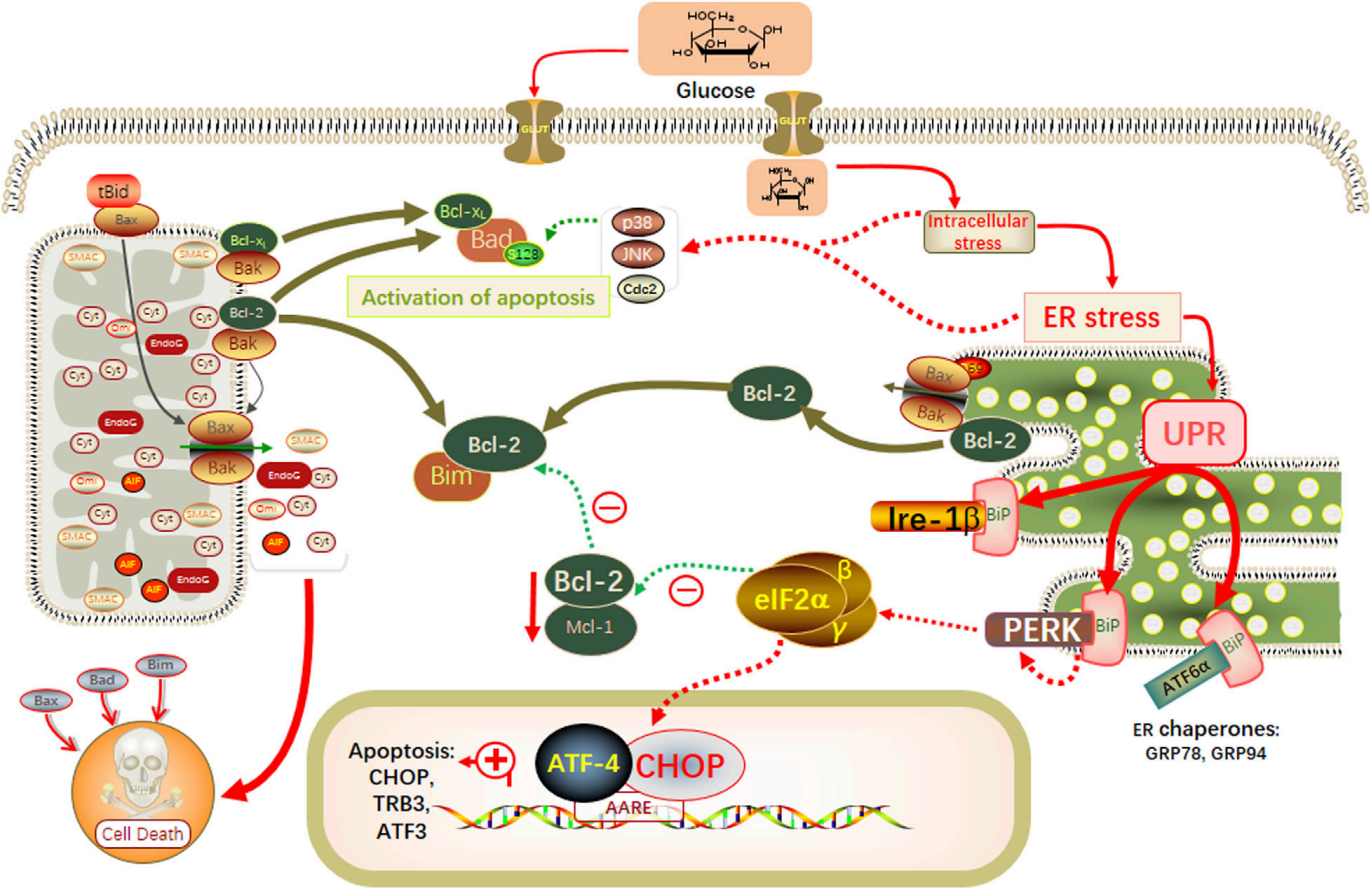
Schematic modeling of the possible underlying molecular mechanisms associated with the therapeutic effects of YSHS on db/db mice.

In this study, through network pharmacology combined with *in vivo* animal experiments and *in vitro* cellular experiments, we demonstrated that YSHS granules modulated the ERS GRP78/CHOP pathway, slowed down the process of hyperglycemia-induced apoptosis, and attenuated the damage of podocytes, which in turn slowed down the progression of DKD (Figure 7).

## Data Availability

The original contributions presented in the study are included in the article/Supplementary Material, further inquiries can be directed to the corresponding authors.

## Abbreviations

Bax, Bcl-2-associated X protein; BCA, Protein Content Assay Kit; Bcl-2, B-cell lymphoma-2; BP, biological processes; CC, cellular components; CHOP, C/EBP homolog protein; DAPA, dapagliflozin; DKD, diabetic kidney disease; ERS, endoplasmic reticulum stress; GO, Gene Ontology; GRP78, glucose regulatory protein 78; HE, hematoxylin and eosin; HG, high glucose; IHC, immunohistochemistry; LDL, low-density cholesterol; MF, molecular functions; PAS, periodic acid–Schiff; PBA, 4-phenyl butyric acid; PBS, phosphate buffer saline; PPI, protein–protein interaction; p-PERK, phosphorylated protein kinase R-like endoplasmic reticulum kinase; Scr, blood creatinine; TC, cholesterol; TEM, transmission electron microscope; TG, triglyceride; UACR, urinary albumin/creatinine; UPR, unfolded protein response; UREA, blood urea; WB, Western blot; YSHS, Yi-Shen-Hua-Shi.
