# Supplementary material for: Exploration of the renoprotective effect of Yi-Shen-Hua-Shi granules on db/db mice and the mechanism of podocyte apoptosis based on the GRP78/CHOP signaling pathway
Source: Front Pharmacol. 2025 Sep 12;16:1586333. doi: 10.3389/fphar.2025.1586333 (PMC12464004; doi:10.3389/fphar.2025.1586333)
Supplement: Supplementary file 1 [file Supplementaryfile1.docx]

**Screening of the optimal dose of YSHS for its protective effect on renal function in db/db mice**

In this experiment, 18 db/db mice were selected and acclimatised and fed until 12 weeks of age, divided into 6 groups of 3 mice each and given gavage treatment for 12 weeks. Experimental administration doses were designed based on human-mouse body surface area conversion (Nair et al., 2018; Nair and Jacob, 2016; Wojcikowski and Gobe, 2014; Yuan, 2004) and references.

The relevant literature on the in vivo dosage of YSHS is as follows: YSHS treatment of diabetic nephropathy in db/db mice was given by gavage of 5, 2.5, and 1.25 g/kg YSHS pellets of the body weight dose to the high-dose, medium-dose, and low-dose groups, respectively (Liang et al., 2022). YSHS regulates Yes-related proteins in mice with diabetic nephropathy, in which the YSHS group was given YSHS by a single daily gavage of 5 g·kg^-1^ (Xiao-fan et al., 2023). In an experimental study in which YSHS regulated the TGF-β1/Smads pathway to ameliorate renal tubular injury in db/db mice, YSHS was given by a single daily gavage of 5 g·kg^-1^ (Li, 2021). Experimental study of YSHS for the treatment of chronic glomerulonephritis in rats was selected for a single dose study (clinically equivalent dose) of YSHS (Zhao et al., 2019). To determine the optimal therapeutic dose of YSHS pellets for attenuating renal injury in the ADR-induced FSGS mouse model, doses of 2, 4 and 8 g/kg of YSHS pellets were administered once daily by tube feeding, and a dose of 4 g/kg body weight was selected based on the results of the UACR was used as the optimal therapeutic dose for the following studies (Tan et al., 2022). For YSHS treatment of IgAN rats, the experimental design was that rats in the YSHS low-dose group and YSHS high-dose group were given YSHS pellets at 1.35 and 5.4 mL/kg/d, respectively (Xu et al., 2024). For YSHS treatment of DKD rats, YSHS was administered by gavage at 2.27 g/(kg· d) in the YSHS low-dose group, and by gavage at 5.54 g/(kg·d) (Han et al., 2023).

The intervention dose of YSHS in the above literature varies widely (1.25 g/kg-8 g/kg) and the dosage varies between rats and mice. In order to determine the optimal intervention dose for our present experiments, we calculated the theoretical dosage of YSHS by using the body surface area conversion method and designed a concentration gradient for pre-testing to screen the dose.

The clinical dosage of YSHS is 1 sachet/dose, 10 g/ sachet, and based on the human-mouse body surface area conversion, the dosage for mice should be 4.7 g/kg per day. Our pretest was designed with 6 drug dose gradients: 0g/kg, 1.175g/kg, 2.35g/kg, 4.7g/kg, 7.05g/kg, and 9.4g/kg. After 12 weeks of intervention treatment, the indexes of UACR, UREA, and Scr were measured. The results of the pre-test showed that YSHS had a tendency to improve renal function as the dose increased, but the improvement of renal function was not significant in mice exceeding the gavage dose of 4.7 g/kg (*P* < 0.05, *P* < 0.01).

**Pretest results：**


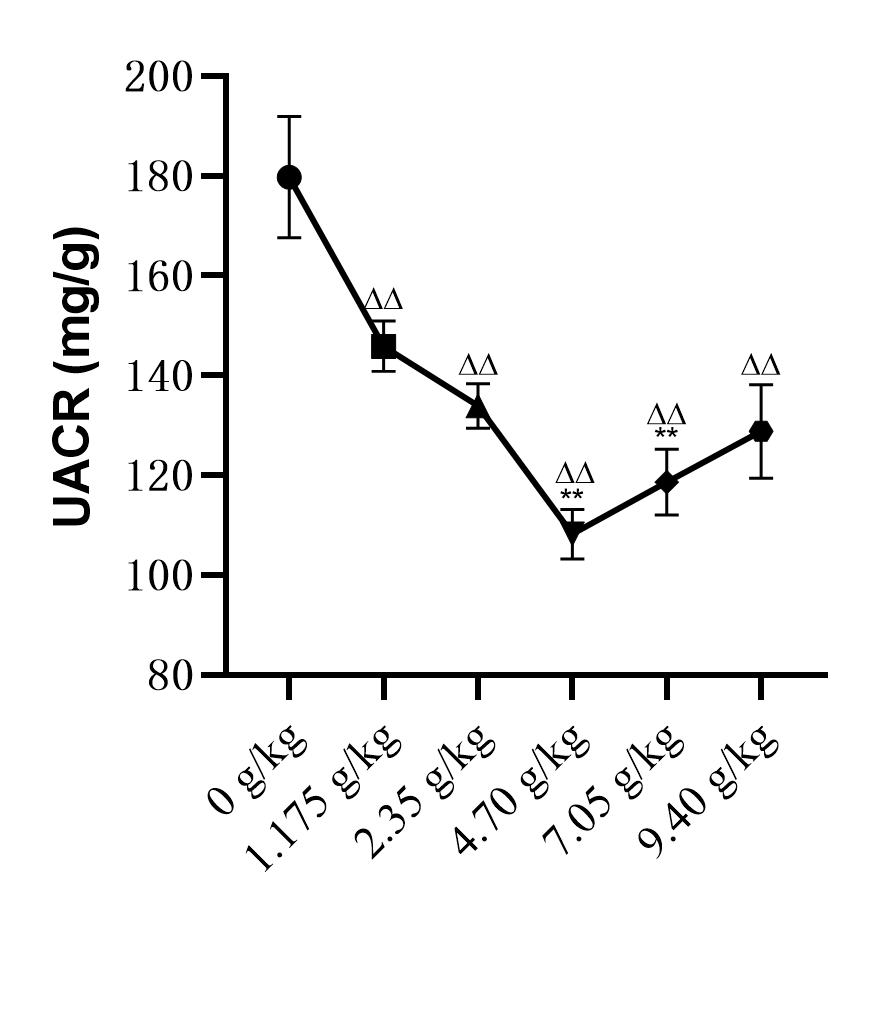


**Figure 1. Effects of different doses of YSHS on UACR in db/db mice**

Note: ^Δ^*P* < 0.05, ^ΔΔ^*P* < 0.01 compared to the 0 g/kg dose; ^*^*P* < 0.05, ^**^*P* < 0.01 compared to the 1.175 g/kg dose; ns: no statistical difference. Data are expressed as *X ± SEM*, *n*=3.


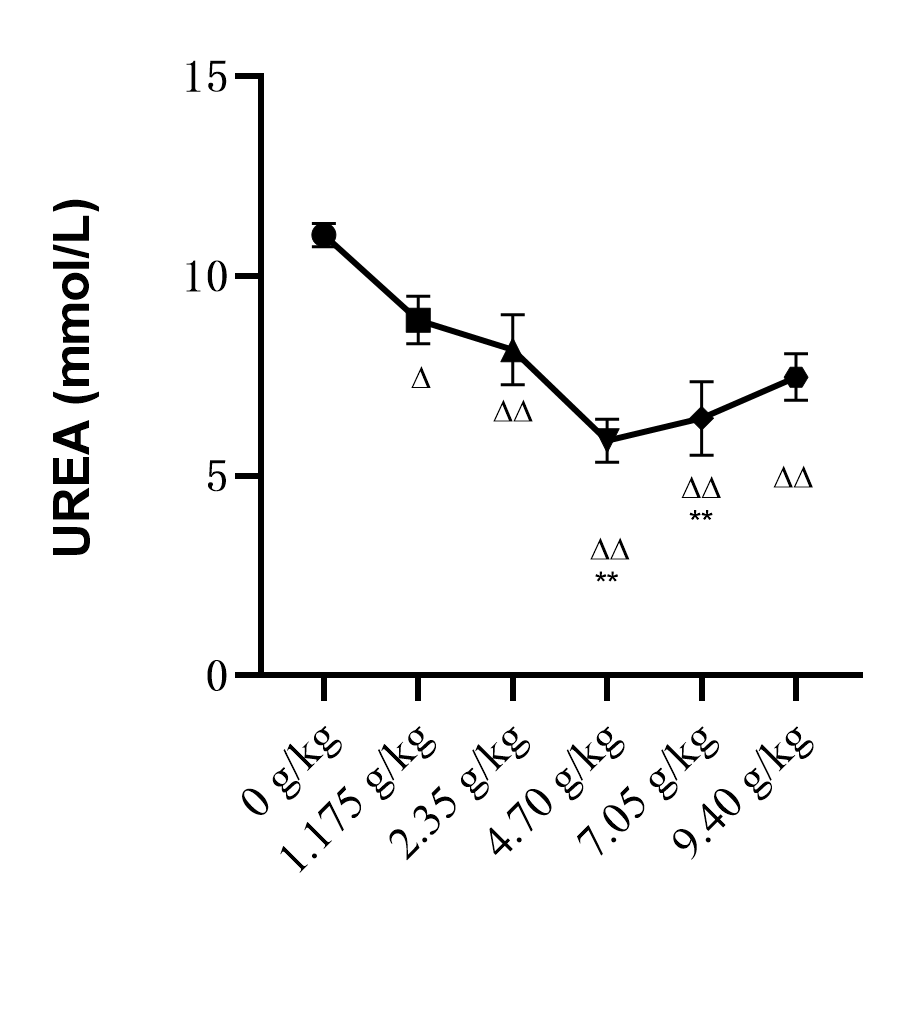


**Figure 2. Effects of different doses of YSHS on UREA in db/db mice**

Note: ^Δ^*P* < 0.05, ^ΔΔ^*P* < 0.01 compared to the 0 g/kg dose; ^*^*P* < 0.05, ^**^*P* < 0.01 compared to the 1.175 g/kg dose; ns: no statistical difference. Data are expressed as *X ± SEM, n*=3.


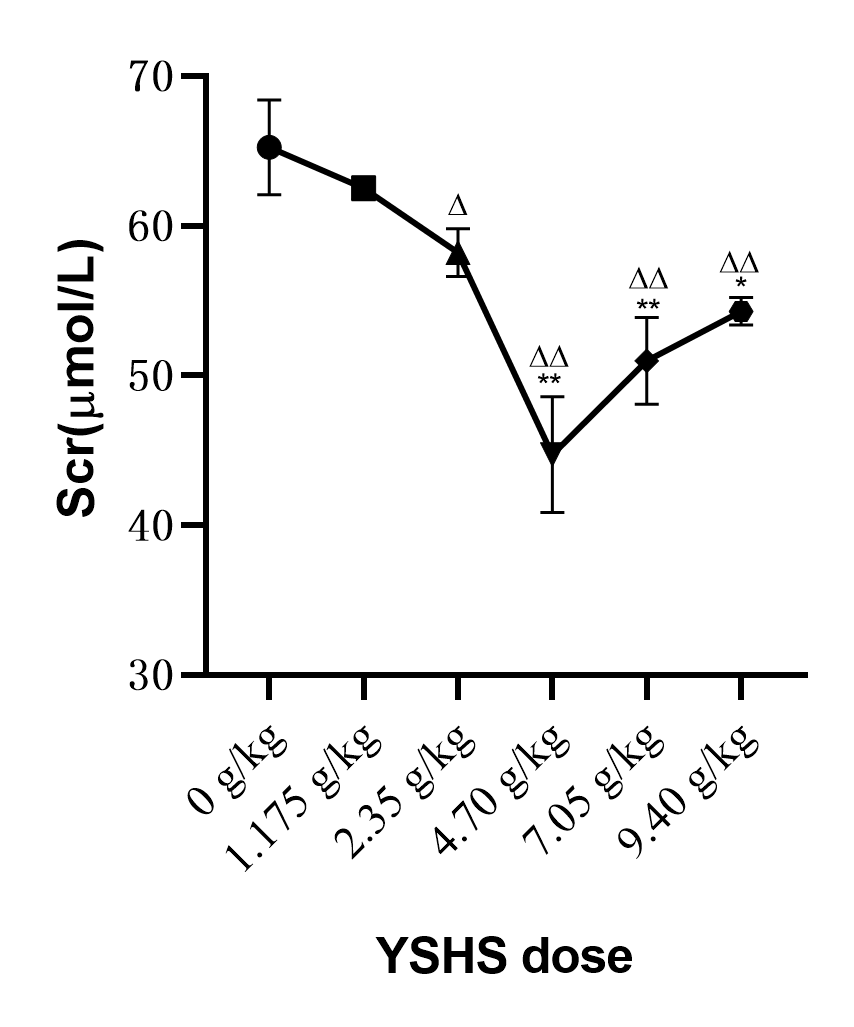


**Figure 3. Effects of different doses of YSHS on SCR in db/db mice**

Note: ^Δ^*P* < 0.05, ^ΔΔ^*P* < 0.01 compared to the 0 g/kg dose; ^*^*P* < 0.05, ^**^*P* < 0.01 compared to the 1.175 g/kg dose; ns: no statistical difference. Data are expressed as *X ± SEM*, *n*=3.

**References:**

Han, C., Shen, Z., Cui, T., Ai, S.S., Gao, R.R., Liu, Y., Sui, G.Y., Hu, H.Z., Li, W., 2023. Yi-Shen-Hua-Shi granule ameliorates diabetic kidney disease by the "gut-kidney axis". J Ethnopharmacol 307, 116257.

Li, C., 2021. Experimental study on the regulation of TGF-β1/Smads pathway by Yi-Shen-Hua-Shi Granules to improve renal tubular injury in db/db mice.

Liang, M., Zhu, X., Zhang, D., He, W., Zhang, J., Yuan, S., He, Q., Jin, J., 2022. Yi-Shen-Hua-Shi granules inhibit diabetic nephropathy by ameliorating podocyte injury induced by macrophage-derived exosomes. Front Pharmacol 13, 962606.

Nair, A., Morsy, M.A., Jacob, S., 2018. Dose translation between laboratory animals and human in preclinical and clinical phases of drug development. Drug Dev Res 79(8), 373-382.

Nair, A.B., Jacob, S., 2016. A simple practice guide for dose conversion between animals and human. J Basic Clin Pharm 7(2), 27-31.

Tan, Z., Si, Y., Yu, Y., Ding, J., Huang, L., Xu, Y., Zhang, H., Lu, Y., Wang, C., Yu, B., Yuan, L., 2022. Yi-Shen-Hua-Shi Granule Alleviates Adriamycin-Induced Glomerular Fibrosis by Suppressing the BMP2/Smad Signaling Pathway. Front Pharmacol 13, 917428.

Wojcikowski, K., Gobe, G., 2014. Animal studies on medicinal herbs: predictability, dose conversion and potential value. Phytother Res 28(1), 22-27.

Xiao-fan, C., Jie-bo, H., Yue, X., Xin-yi, F., Yue-yi, D., Yi-fei, Z., Hui-juan, W., 2023. Ｒegulation of Yes associated protein in diabetic nephropathy

mice by Yishen Huashi Granule. Chinese Pharmacological Bulletin 39(10), 1944-1949.

Xu, R., Zhang, J., Hu, X., Xu, P., Huang, S., Cui, S., Guo, Y., Yang, H., Chen, X., Jiang, C., 2024. Yi-shen-hua-shi granules modulate immune and inflammatory damage via the ALG3/PPARγ/NF-κB pathway in the treatment of immunoglobulin a nephropathy. J Ethnopharmacol 319(Pt 2), 117204.

Yuan, H.J.H.H.X.-H.C.Z.Y.Z.Q.S.S.R., 2004. Dose conversion among different animals and healthy volunteers in pharmacological study. Chin J Clin Pharmacol Ther(09), 1069-1072.

Zhao, J., Chan, Y.C., He, B., Duan, T.T., Yu, Z.L., 2019. A patent herbal drug Yi-Shen-Hua-Shi granule ameliorates C-BSA-induced chronic glomerulonephritis and inhabits TGFβ signaling in rats. J Ethnopharmacol 236, 258-262.
